# Incorporating industrial design pedagogy into a mechanical engineering graphics course: a discipline-based education research (DBER) approach

**DOI:** 10.1186/s40594-018-0122-7

**Published:** 2018-07-04

**Authors:** Sunni Newton, Meltem Alemdar, Ethan Hilton, Julie Linsey, Katherine Fu

**Affiliations:** 1 Center for Education Integrating Science, Mathematics, and Computing, Georgia Institute of Technology, 817 W. Peachtree Street, NW, Suite 300, Atlanta, GA 30308 USA; 2 The George W. Woodruff School of Mechanical Engineering, Georgia Institute of Technology, 801 Ferst Drive, Atlanta, GA 30332-0405 USA

**Keywords:** Engineering graphics, Design, Discipline-based education research (DBER)

## Abstract

**Background:**

A redesigned curriculum for teaching engineering graphics was adopted in an introductory mechanical engineering course. The rollout of this curriculum was staggered, allowing for comparisons of student perceptions across the newly revised and previous instructional approaches. The new curriculum borrows from content and pedagogy traditionally employed in industrial design courses. The discipline-based education research (DBER) framework was used to investigate the manner in which the new curriculum was implemented and student reactions to this change. By using this approach, the researchers were able to incorporate and emphasize the unique aspects of the subject matter itself, as well as the attributes of the engineering discipline in which the course was embedded.

**Results:**

Results indicated that students exhibited positive reactions to the sketching instruction, as well as various other aspects of the course, and that reactions were generally more positive among students in the redesigned course.

**Conclusions:**

The contributions of this paper are twofold: illustrating the application of a specific research framework and providing results of an investigation of a redesigned curriculum. The redesigned curriculum was generally received well by students, and the partnership between the education researchers and faculty proved fruitful in allowing for nuanced investigation of the course redesign. Practical considerations for undertaking this type of research are also outlined.

## Background

Over the past several decades, there has been substantial growth in the breadth of scholarly work aimed at improving undergraduate science, technology, engineering, and math (STEM) education through the application of principles of human learning, specifically via use of the discipline-based education research (DBER) framework (Borrego and Bernhard 2011; Fortenberry et al. 2007; Froyd and Lohmann 2014; National Research Council 2012; Talanquer 2014). Recognizing DBER’s importance in improving undergraduate science education, scholars have conducted a wide range of DBER studies, ranging from basic (e.g., cognitive processes involved in student misconceptions) to applied research (e.g., application of instructional technologies) (National Research Council 2012). A formal research framework, DBER, rests on this principle: that when expertise on human learning principles and research methods is combined with a nuanced understanding of the discipline being researched, meaningful conclusions about student experiences and learning within that discipline can be reached (National Research Council 2012). The increased interest in DBER also promotes collaboration between educational researchers and core science (e.g., engineering) scholars. The nature of this collaboration helps to identify and measure knowledge of best instructional approaches.

An opportunity to engage in DBER arose through the authors’ collaborative work in investigating changes to a curriculum redesign in the school of mechanical engineering (ME) within a US institution of higher education. The utilization of DBER in investigating curriculum redesign efforts is not a common practice. Institutional change efforts involving engineering education partnerships, both at the K-12 and higher education levels, were one of several foci of a National Science Foundation (NSF) funded grant. Specifically, the institutional change effort being assessed here was a curriculum redesign occurring in an introductory mechanical engineering course titled “Introduction to Engineering Graphics” (IEG). The course introduces students to engineering graphics and design with a focus on sketching techniques and computer-aided design (CAD) software.

In this introduction, we will provide background on DBER in general, review the key components of the course and its role in the broader engineering curriculum, describe the specifics of the curriculum redesign efforts, and discuss our unique experience with DBER and the various roles played by the educational researchers and the mechanical engineering faculty members in the redesign.

The purpose of this paper is to demonstrate the use of the DBER framework to investigate the curriculum design changes to an introductory mechanical engineering course. This framework engages educators, subject matter experts, and educational researchers in collaborative and iterative design of an undergraduate course.

### Discipline-based educational research (DBER): overview and relevance

At the core of DBER is the recognition that educational research methods are not a “one size fits all” endeavor. Training in educational research methodology and statistical analysis is necessary but not sufficient for the successful enactment of DBER; a high degree of core content knowledge is also required. DBER is “distinguished by an empirical approach to investigating learning and teaching that is informed by an expert understanding of disciplinary knowledge and practice” (National Research Council 2012, p. 11). This “expert understanding” may lie within the researcher him/herself, as is the case for scholars who possess both educational research acumen and deep technical knowledge. Or a researcher may seek out this “expert understanding” by collaborating with a teacher or professor in the focal discipline. In either case, a deep understanding of the discipline is central to success in DBER.

The team of researchers carrying out this project was comprised of one graduate student, two educational research experts, and two “content” experts in the areas of design, creativity, sketching and CAD. Because of the highly specific nature of the curriculum redesign efforts undertaken in the IEG course, coupled with the educational researchers’ relatively modest technical content knowledge of engineering sketching and CAD techniques, DBER was selected as the appropriate framework for an assessment of the curriculum redesign. A high level of collaboration between the educational researchers and the content experts was vital to the successful execution of this research, and DBER is ideally suited to this collaborative approach. The purpose of this research was to understand the extent to which different instructional methods are associated with student perceptions of learning, utility, efficacy, experience, and future use of these course topics. The National Research Council (NRC) recently released a report on DBER within the context of undergraduate science and engineering (National Research Council 2012). In this report, they outlined a series of recommended areas of inquiry for engineering-related DBER, several of which are directly addressed by this project: “the extent to which engineering faculty adopt evidence-based practices”, “the extent to which faculty take a scholarly approach to teaching and learning”, and “the extent of collaboration with higher education researchers, learning scientists, and other scholars of teaching and learning” (National Research Council 2012, pp. 47–48).

Educational research focused specifically on engineering instruction at the higher education level has emerged as a robust area of inquiry over the past few decades, with dedicated journals (e.g., *Journal of Engineering Education* (2018); *International Journal of Engineering Education* (2018)), conferences (e.g., Educational Research and Methods Division of the American Society of Engineering Education (“American Society for Engineering Education, Educational Research and Methods Division"” 2018)), and graduate training programs (e.g., Department of Engineering Education at Virginia Tech (“Virginia Tech Department of Engineering Education” 2018); School of Engineering Education at Purdue University (“Purdue Engineering Education Department” 2018)) being created to support the emergent field (Fortenberry et al. 2007; Froyd and Lohmann 2014; National Research Council 2012). This area of inquiry underwent an increased formalization beginning in the late twentieth century and passing a “tipping point” around the mid-2000s; integral to this formalization is a more rigorous, evidence-based approach to studying how students learn, as opposed to more anecdotal, ad hoc, and experience-based methods that often previously guided the evaluation of and decisions around instructional change efforts (Felder et al. 2005; Johri and Olds 2011; Froyd and Lohmann 2014).

Recent examples of DBER work in engineering reflect a variety of research efforts, including developing concept inventories for engineering topics (Reed-Rhoads and Imbrie 2008), assessing engineering students’ misconceptions related to thermodynamics and heat transfer (Prince et al. 2010), and conducing a meta-analysis of studies on the nature of engineering students’ knowledge (Turns et al. 2005). Various labels have been used to describe DBER directed at the engineering field, including engineering education research (EER) and rigorous research in engineering education (RREE); regardless of the scholars’ preferred terminology, such work reflects the requirement that both a deep understanding of the discipline’s content and practices and theory from the learning sciences undergird the research design and interpretation (Borrego and Bernhard 2011; Streveler and Smith 2006).

Such research efforts are aimed at addressing both the educational requirements of and areas where improvement is needed in the broad field of engineering: promoting diversity among members of the profession, improving the “public image” of engineering and the nature of the public’s understanding of what engineering is and what engineers do, maximizing engineering students’ ability to tackle the world’s large and complex problems, and understanding how learning in the discipline occurs in order to improve the learning process (Borrego and Bernhard 2011). The intent and goals of such efforts are effectively summarized in the following quote: “We want to understand how students learn engineering. It is our hope that by supporting fundamental research, we can better understand how to create a more innovative, efficient, and enticing engineering curriculum that can attract a more talented, innovative, and diverse student body” (Borrego and Bernhard 2011, p. 21; Gabriele 2005, p. 286).

### Introduction to engineering graphics (IEG): course basics

The IEG course is the introductory course in the required design sequence for mechanical engineering majors and aerospace engineering majors. The class is taken primarily by first-year students. In some cases, transfer students and aerospace engineering students take the course later in their undergraduate tenure. Students in the course are mostly mechanical engineering and aerospace engineering majors; some students majoring in industrial design or materials science and engineering take the course as an elective. The main skill sets covered in the course are engineering visualization, sketching, and CAD.

**Fig. 1:**
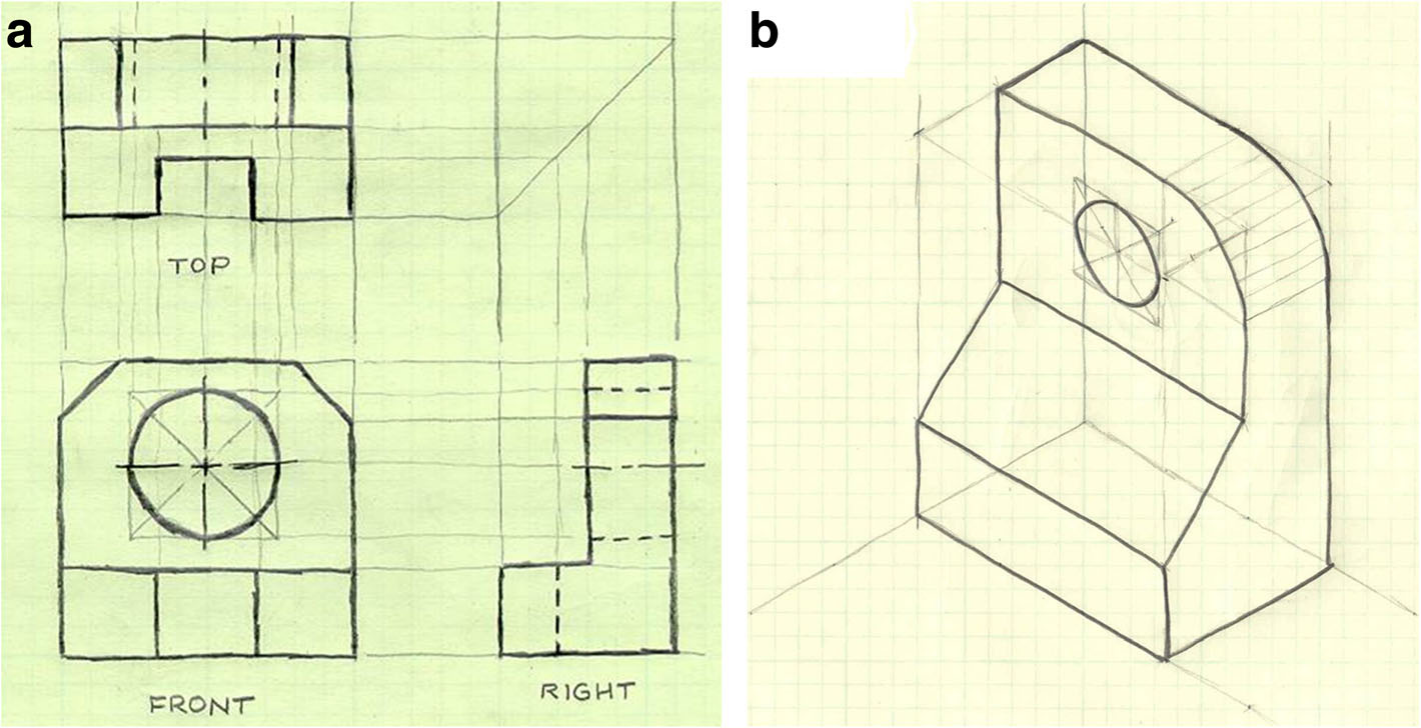
Example of exercises used to teach sketching in IEG course before the update to the industrial design pedagogy. **a** Sketching multiple views of an object. **b** Sketching an isometric view of an object

Generating visual representations through sketching and CAD is a key skill for engineers (Dym et al. 2005). These representations provide collaborators with a common mental model (Goldschmidt 2007) and can be used as early-stage prototypes (Yang 2004). Recognizing the importance of students’ ability to generate visual representations, the IEG course within our institution’s school of mechanical engineering was revamped in 1999 using a backward design approach and included both sketching and the use of CAD programs (Pucha and Utschig 2012). The course included instruction in developing both 2D and 3D models. Figure 1 shows exercises that students completed during the sketching portion of the course.

**Fig. 2:**
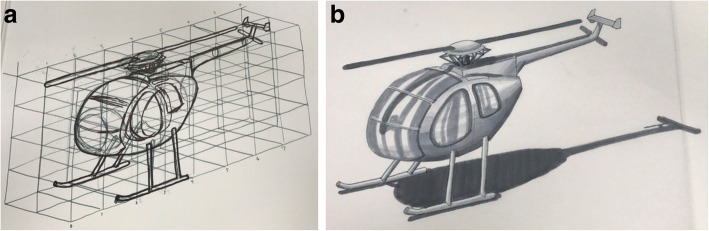
Student submission for lab assignment requiring a sketch in perspective view. **a** Draft sketch with scaffolding. **b** Final composition sketch with shading and shadows

Sketching has been found to improve product design outcomes of a team when used early in the design process (Song and Agogino 2004; Yang 2009). Learning sketching has also been shown to improve spatial visualization, a key skill in engineering (Sorby 2009). For these reasons, the course was updated to include instruction in sketching based on pedagogy commonly found in industrial design courses (Hilton et al. 2016). This version of the course maintained the same instructional content on CAD programs, but updated the portion of the class devoted to sketching to include techniques such as shading and sketching in perspective as seen in the student submission shown in Fig. 2. These techniques allow students to sketch more realistic renderings of an object and are intended to enhance their visualization skills. It is important to note that the sketching instruction emphasizes skill building for visual communication, rather than for the aesthetic fine art quality of the renderings.

**Fig. 3:**
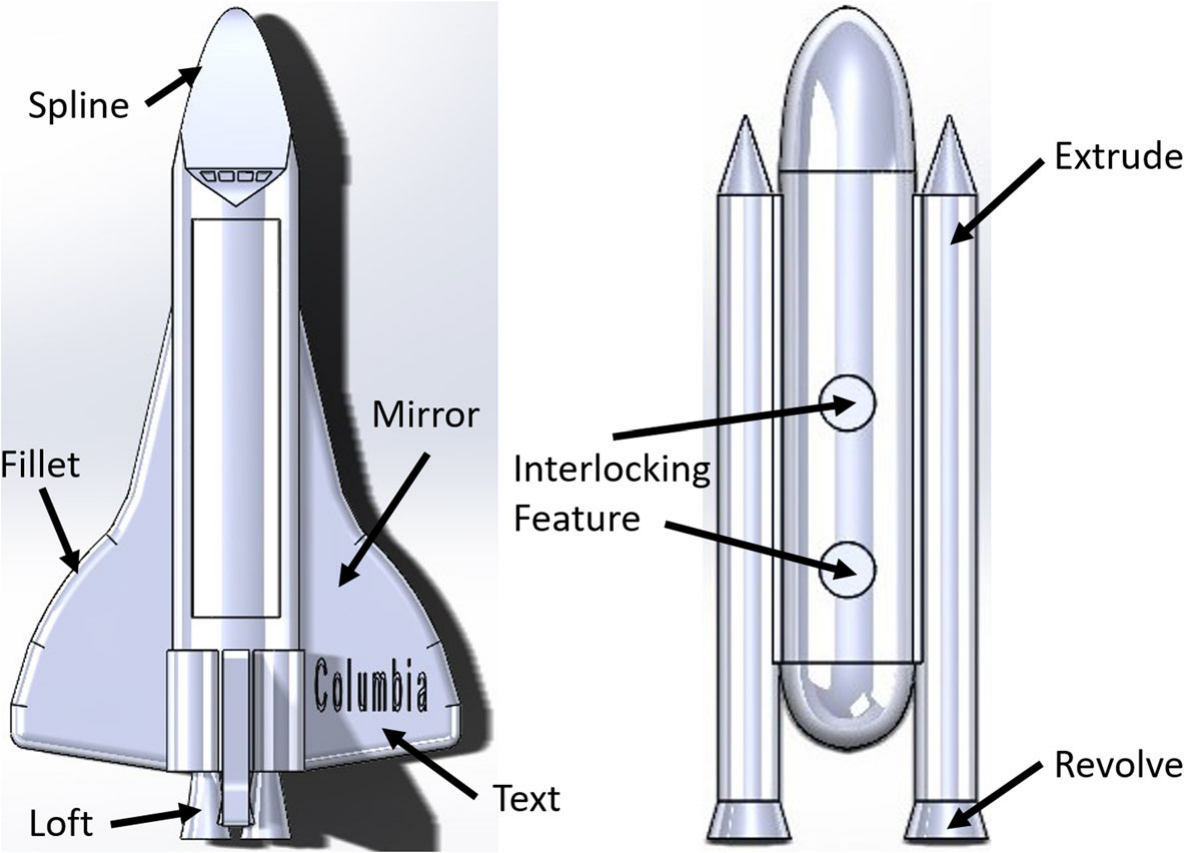
Student submission for the individual project assignment

By virtue of its high level of required project work, carried out both by individual students and teams of students, the course provides students with the opportunity to develop their skills in the areas of teamwork and written (e.g., reports) and verbal (e.g., presentations) communication skills. The first project assigned in the redesigned course (referred to as the individual project) requires students to create models of objects in CAD, which in some cases will be printed on a 3D printer (not all projects are able to be 3D printed due to time and resource constraints). This project utilizes students’ CAD skills, while engaging their creativity to model objects of their own design, which must include at least one interlocking feature with another part and four different major CAD operations or feature types. Objects selected for these individual projects range from phone holders for students’ bikes to small toy cars and space shuttles such as the one seen in Fig. 3. Concepts of tolerance analysis and design for manufacturing are integrated into this project through analyzing how the two pieces would fit together.

**Fig. 4:**
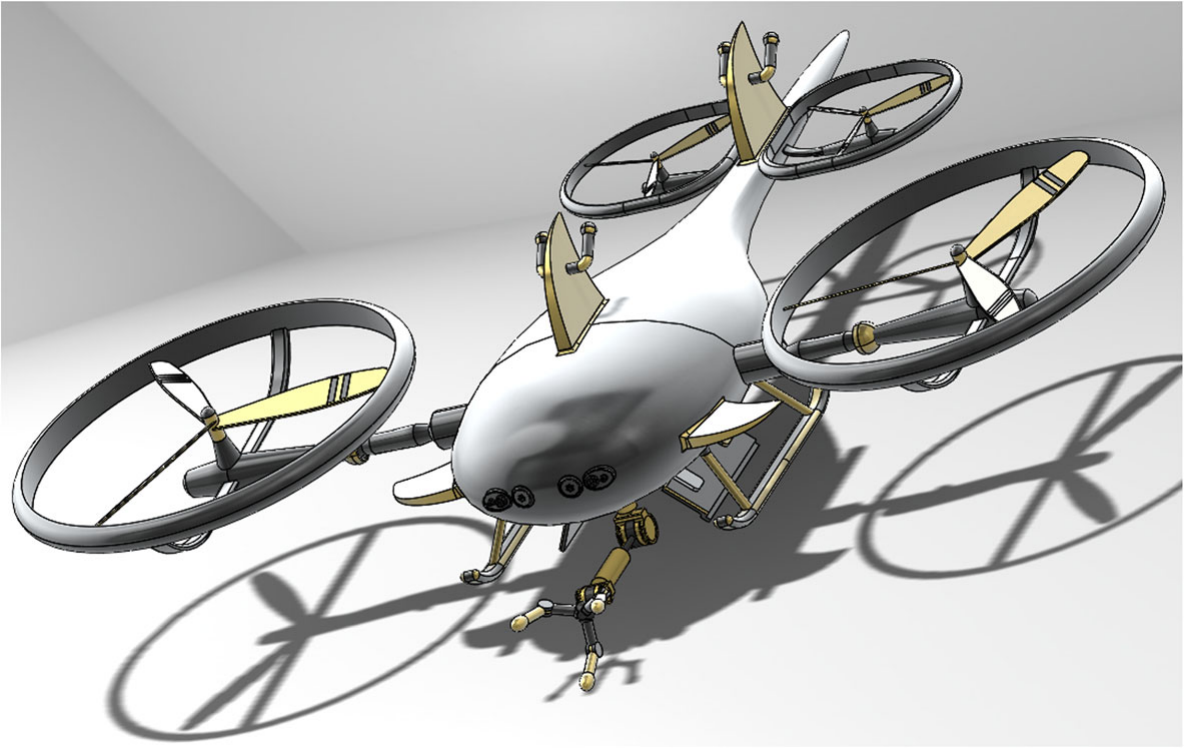
Student submission for the team project assignment

The second project assigned in the redesigned course (referred to as the team project) requires teams of four to six students to create a complex assembly of at least 15 separate complex parts. Past projects have included fantasy objects like Dr. Who’s screwdriver, robots, concept cars, and airplanes. Past projects have also involved new inventions, such as a record player that plays disks vertically and a vehicle that flies and dives into the ocean (as seen in Fig. 4). Because both projects and the sketching assignments allow students to select their own items to design, the class provides students an opportunity to exercise and develop their design creativity. With its emphasis on engineering visual communication, sketching, and CAD, this course sets the stage for the next course in the design sequence, Creative Decisions and Design. The Creative Decisions and Design course further develops students’ design capabilities and technical skills in engineering design by engaging them in a team design and competition project, manufacturing processes, and mechatronics.

The other key objective of the course is to inform students about mechanical engineering: what does their selected major entail, what does it look like in the real world? Instructors in the course accomplish this objective via the 3D printing of students’ individual projects, brief discussions of engineering design processes, products and the associated manufacturing processes, and the team project components of the course. Prior research has shown that student development toward a “practitioner” level of performance is aided by engagement in tasks that make content “real” and serve as authentic activities (Bhattacharyya and Bodner 2014). By providing a variety of tasks and experiences that are intended to be authentic, positive, and engaging, the course aims to reinforce students’ major selection and move students closer to “practicing” engineers.

### Introduction to engineering graphics (IEG): course redesign

The key purpose of this research is twofold: (1) to investigate the iterative changes to the IEG course carried out by the course instructors in their curriculum redesign efforts and (2) to examine student perceptions of the redesigned curriculum. The key distinctive elements of the new approach are (a) an emphasis on industrial design sketching elements, as opposed to solely focusing on engineering-related sketching elements, (b) the use of in-class critiques for sketching evaluation and feedback delivery, of the type frequently used in design and architecture courses, and (c) the inclusion of rapid prototyping via 3D printing of students’ individual projects. The new approach also entailed changes in the way the CAD instruction was carried out, with the primary of these changes being the adoption of a “semi-flipped” classroom design in which CAD content instruction was delivered outside of class via a series of online video modules and class and lab time were used for hands-on practice with the CAD software. We call the design “semi-flipped” because some lecture content was still delivered during class time in order to connect CAD to manufacturing applications, and to introduce students to geometric dimensioning and tolerancing (GD&T), which are not covered in the online video modules.

During the Fall 2015 semester, three of the four IEG course instructors integrated the new sketching instruction and in-class critiques into the course. These sections are considered the experimental sections (five sections in total). The fourth instructor taught sketching in the format used prior to the course redesign, which focused solely on engineering drawing with no industrial design emphasis or in-class critiques; as such, those sections are considered the control sections. The course revision sought to achieve multiple goals, including making the class more engaging to students, increasing the connection of the class to real-world applications of engineering, increasing the opportunity to be creative by increasing the level of student choice included in the projects, and improving students’ sketching confidence and abilities. The individual project was specifically intended to address the goal of increased student engagement, as it illustrates the engineering rapid prototyping process of designing objects in CAD and 3D printing them (Hilton et al. 2016).

The final team design project was modified to allow students to work on any assembly or product they desired. Modifications were made to project requirements to ensure students had to pick sufficiently complex projects; furthermore, the instructor reviewed and approved all projects prior to students beginning work. The ability to choose any product within the constraints of the projects allows students to better engage with their own interests and to illustrate the wide variety of products that mechanical and aerospace engineers design. The redesigned curriculum allows for self-directed learning in that students are free to choose, with some constraints, what they sketch in some sketching assignments and what they design in the individual and team projects. The emphasis on student choice was intended to both improve student engagement and enhance the level of creativity elicited from students throughout the course assignments.

The instructors, based on the design research literature, also recognized that free-hand sketching is critical to communication in engineering (Goldschmidt 2007; Shah et al. 2001) and supports reasoning in engineering (Cross and Roy 1989; Gobert and Clement 1999). The goals of introducing industrial design-based sketching were as follows: to increase student sketching confidence and skill, to enhance students’ ability to communicate visually, and to encourage students to be more willing to use sketching for visual communication in later engineering classes.

During the Spring 2016 semester, all instructors adopted the new sketching approach in at least some capacity (one of the three original instructors who taught the new approach during Fall 2015 did not teach the course during Spring 2016). For this semester, rather than having an experimental vs. control group comparison, we investigated potential differences in student experiences with instructors who had previously taught the new sketching approach (“veteran instructors”) vs. the instructor who was teaching the new sketching approach for the first time in Spring 2016 (“new instructor”). The results from the Fall 2015 and Spring 2016 data collection efforts, both of which included a sketching survey conducted at the conclusion of the sketching portion of the course and an end of semester survey, are presented below in the results section. A similar set of data was collected in the Spring 2015 semester, but with the intention of being used formatively; as such, these data are not included here.

### DBER research in IEG course

The educational researchers (a subset of the authors of this paper) who evaluated the course redesign were entirely external to the group who originally conceptualized, introduced, and carried out the curriculum redesign. It has been noted by DBER experts that, in many cases, curriculum innovation research involves assessment of an innovation by the instructor/researcher who created the innovation, a scenario in which bias can be introduced (National Research Council 2012). These authors note that “one approach to counter this bias is to study other instructors who are implementing the innovation in question. However, it can be difficult to recruit others to teach specific course content in specific ways, independently of the research team” (National Research Council 2012, p. 54). The arrival of a new IEG course instructor on campus prior the Fall 2015 semester, one who was previously unfamiliar with the curriculum and was taught to implement the new sketching instructional methods, allowed for exactly this scenario. And by being totally external to the ME curriculum redesign efforts, the educational researchers approached the research efforts with little attachment to a given outcome.

The following research questions are investigated in this project:

1. How do students react to the curriculum redesign?
2. Do students’ perceptions of the curriculum differ, and if so how, between those in the redesigned curriculum and those in the standard curriculum?
3. Do students’ perceptions of the curriculum differ, and if so how, on the basis of their instructors’ experience level with teaching the redesigned curriculum?

One of our goals in this project, other than the primary research goal of assessing students’ perceptions of the curriculum redesign, was to investigate and define the specific components of the course redesign and to discern instructors’ plans for implementing them, including timelines and specific lesson plans. This was also critical for properly designing the research instruments. To accomplish this goal, the education researchers met multiple times with the instructors prior to and during implementation of the curriculum redesign. A key outcome of these meetings, and one of our main accomplishments in this project, was facilitating the sharing of information and instructional strategies among instructors. Meetings were organized to facilitate collaboration among instructors (e.g., sharing of ideas and resources, identifying and capitalizing on each others strengths). Instructors also held weekly meetings to facilitate course planning, training, and resource sharing on their own.

Another beneficial outcome associated with this research was further collaboration between the educational researchers on the project and one of the IEG instructors on a separate line of creativity research the instructor was interested in pursuing. Students in one instructor’s IEG sections were instructed on several novel ideation methods, with the aim of enhancing their design creativity, specifically within the context of selecting objects to model for their individual projects. We were able to expand ongoing data collection opportunities to investigate student perceptions and use of these ideation methods, resulting in student feedback that the instructor then used to iterate on the ideation method instruction in later semesters (Pucha et al. 2017; Pucha et al. 2016).

As the work progressed, the initial scope expanded to include the following goals:

1. To promote the application of our findings to inform overall acceptance or rejection of classroom innovations, and also smaller tweaks to these innovations, informed both by our research results and by the interactions promoted among instructors as a result of their engagement in this research
2. To analyze the experience of engaging in DBER as an external research entity within the context of a multi-instructor, multi-section mechanical engineering course with a long-established instructional methodology, a mix of tenure-track and non-tenure-track instructors, and a departmental-level request for innovation within the course

## Methods

### Research design

This research uses a quantitative approach, employing surveys with Likert type, closed-ended items intended to determine the nature of students’ experiences with the IEG course.

### Participants

Participants in this study were students enrolled in the IEG course during either the Fall 2015 or the Spring 2016 semester. All students enrolled in the course were invited to participate in this research under a protocol approved by the Institutional Review Board. Across the four total surveys administered (two each during the Fall 2015 and Spring 2016 semesters), between 136 and 354 students responded to a given survey, representing response rates ranging from 40.5 to 89.2%. It should be noted that the markedly lower response rate of 40.5% is associated with only one survey (all others were above 75%), which researchers attribute to not having administered the survey during class time. This was our first and only attempt to invite students to take the survey via e-mail; the low response rate suggested that this was not effective, and all subsequent surveys were introduced and administered during class.

Across the surveys, most students (72.1–78.6%) were male. A small number of students either left this item blank or indicated that they preferred not to answer. The gender composition of student respondents remained stable across survey administrations. Across the surveys, most students (61.6–73.5%) were mechanical engineering majors. The only other major represented by a sizable portion of students (18.4–36.2%) is aerospace engineering (AE). The “other” category includes students with other majors or double majors, most of which include ME or AE. A handful of students did not respond to this item. The major composition of students remained stable across survey administrations.

### Data sources

#### Sketching survey

The sketching survey contained 23 items and was administered via the online survey software SurveyMonkey (SurveyMonkey Inc 2015-2016) to students in all IEG sections. The informed consent form was integrated into the beginning of the survey, in place of obtaining written consent on paper. The goal of this survey was to collect students’ perceptions of and opinions regarding the sketching portion of IEG. The survey was comprised of locally developed items, which were written by the educational researchers and reviewed and revised by all IEG instructors, who in some cases proposed new items. A 6-point response scale ranging from “Strongly Disagree” to “Strongly Agree” was used. The survey, which took roughly 15 min to complete, was given immediately after the sketching portion of the course, roughly 5–6 weeks into the semester, during both the Fall 2015 and Spring 2016 semesters.

#### End of semester survey

The end of semester survey contained 49 items and was administered via the online survey software SurveyMonkey (SurveyMonkey Inc 2015-2016) to students in all IEG sections. The survey was designed to assess student perceptions of various aspects of the course, including how the sketching instruction had continued to serve them later in the semester, the CAD instruction, and their individual design and team projects. The survey was comprised of locally developed items, which were written by the educational researchers and reviewed and revised by IEG instructors, who in some cases proposed new items. A 6-point response scale ranging from “Strongly Disagree” to “Strongly Agree” was used. The survey, which took roughly 15 min to complete, was given during the last week of the course, during both the Fall 2015 and Spring 2016 semesters.

Throughout the course of this research, our efforts expanded to address specific topics of interest to one of the course instructors. This instructor taught ideation methods intended to enhance creativity to some of his sections; students in these sections were also asked additional items about their experiences with the ideation methods. These data are published elsewhere (Pucha et al. 2017; Pucha et al. 2016), and as such these items and corresponding results are not included here.

### Data analysis

Quantitative data were analyzed with a combination of descriptive statistics, to capture overall trends in the data, and independent samples *t* tests, to determine the presence of group differences, either between experimental and control groups, or between new and veteran instructor groups.

## Results

As discussed in the data sources section, data were collected with two separate instruments, the sketching and end of course surveys, in two semesters, Fall 2015 and Spring 2016. The Fall 2015 data allow for comparisons between the experimental and control groups, whose instructors implemented the new and traditional instructional approaches, respectively. The Spring 2016 data allow for comparisons between the veteran instructor group and new instructor groups, whose instructors had and had not taught the new instructional approach prior to that semester, respectively. Descriptive statistics were calculated, and independent samples *t* tests were conducted to compare means across the relevant student groups within each semester.

### Sketching survey

Results for the sketching survey are shown in Table 1. Across both semesters, mean responses on all items for the experimental and veteran instructor groups, and mean responses on most items for the control and new instructor groups, were at or above a value of 3.5, indicating a mean response on the positive side of the response scale. So generally, students view the sketching instruction favorably.

**Table 1:** Sketching survey results (Fall 2015 and Spring 2016)

Survey item	Fall 2015							Spring 2016						
	Control			Experimental			*t* value	New			Veteran			*t* value
	*N*	Mean	SD	*N*	Mean	SD		*N*	Mean	SD	*N*	Mean	SD	
The format of the in-class critiques was appropriate for this course	n/a			197	4.98	0.74	n/a	n/a			191	4.87	0.81	n/a
I felt comfortable speaking about the work of others during the in-class critiques	n/a			197	4.75	0.91	n/a	n/a			191	4.52	1.03	n/a
I felt comfortable speaking about my own work during in-class critiques	n/a			197	4.54	1.14	n/a	n/a			191	4.60	1.04	n/a
The in-class critiques enhanced my ability to learn how to sketch	n/a			197	4.53	1.17	n/a	n/a			191	4.32	1.17	n/a
Participating in the sketching portion of this course improved my ability to sketch	139	4.74	1.27	197	5.41	0.79	5.51*	163	5.02	1.01	191	5.18	0.80	− 1.72
Participating in the sketching portion of this course improved my ability to communicate a specific idea to a specific audience with my sketches	139	4.29	1.16	197	4.95	0.89	5.69*	163	4.55	1.01	191	4.84	0.88	− 2.87*
Participating in the sketching portion of this course improved my ability to prepare a set of working drawings for manufacturing	139	4.71	1.22	197	4.74	1.03	0.19	163	4.88	0.98	191	4.84	0.92	0.45
Participating in the sketching portion of this course increased my creativity	139	3.58	1.36	196	4.32	1.12	5.22*	163	3.72	1.28	191	4.20	1.12	− 3.76*
The sketching portion of this course was a good use of my time	138	3.57	1.41	197	4.51	1.14	6.53*	162	3.75	1.30	191	4.42	1.11	− 5.24*
I feel more confident in my sketching ability as a result of participating in the sketching portion of this course	138	4.50	1.30	197	5.06	1.02	4.20*	163	4.67	1.11	191	4.90	0.99	− 2.02*
I believe the sketching training I received in this course will serve me well in later engineering courses	139	4.30	1.29	197	4.77	1.16	3.48*	163	4.40	1.24	191	4.77	1.01	− 3.05*
I would have preferred to move straight into CAD instruction without spending class time on hand sketching	135	3.76	1.48	197	3.16	1.47	− 3.60*	163	3.52	1.48	191	3.36	1.38	1.05
I felt that the grading of the sketching portion of the course was fair	135	4.00	1.35	197	5.01	1.12	7.16*	163	4.75	1.08	191	4.60	1.15	1.32
I felt that the peer feedback process was effective in improving the quality of my work on lab assignments	134	3.32	1.31	n/a			n/a	163	3.50	1.27	191	4.03	1.13	− 4.16*
I felt that the first round of TA feedback was effective in improving the quality of my work on lab assignments	134	4.16	1.39	n/a			n/a	162	4.43	1.1	191	3.91	1.14	4.29*
I believe I will sketch more frequently (when it’s not directly assigned) in this and later ME courses as a result of participating in the sketching portion of this course	135	3.46	1.33	197	4.13	1.28	4.61*	163	3.53	1.22	190	3.91	1.20	− 2.93*
I believe the sketching training I received in this course will enhance my ability to use CAD	135	3.99	1.27	197	4.20	1.19	1.59	163	3.99	1.21	191	4.11	1.20	− 0.91
I believe the sketching training I received in this course improved my visualization skills	135	4.26	1.21	197	4.67	1.04	3.26*	163	4.26	1.14	191	4.68	1.07	− 3.56*
I enjoyed the sketching portion of this course	135	3.43	1.54	197	4.67	1.04	6.01*	163	3.58	1.52	191	4.27	1.32	− 4.48*

*n/a* not applicable

*Statistical significance of *p* < .05

For the subset of survey items common to both sections, *t* tests were run to investigate differences between the relevant groups; the results of these comparisons are provided in Table 1. On most items, a significant difference was present (*p* < .05), and nearly all differences were in the direction of the experimental group or the veteran instructor group providing a more positive mean response than did the control group or the new instructor group. Taken together, these results suggest that teaching the new instructional approach to sketching, and being more experienced with the new instructional approach, are each associated with more favorable student perceptions of the sketching instruction. As indicated in the literature, learning sketching is associated with improved spatial visualization, which is a highly valued skill in engineering (Sorby 2009). It is possible that students viewed the redesigned sketching instruction favorably because they found this approach to sketching to be a useful skill that supported their overall learning in the class.

In the Fall 2015 data, only two items failed to show a significant difference (*p* < .05) between groups: “Participating in the sketching portion of this course improved my ability to prepare a set of working drawings for manufacturing” and “I believe the sketching training I received in this course will enhance my ability to use CAD”. These results indicate that the two sketching instructional approaches were comparably effective on some of the more technical aspects of sketching. On all other aspects of the sketching instruction on which students were surveyed (e.g., improved ability to sketch, improving communication, improving creativity, being a good use of students’ time, and being fairly graded), the new approach was perceived as significantly more effective.

It is interesting to note that in the Spring 2016 data, on what is arguably the most important objective of the sketching instruction, improving students’ ability to sketch, the new and veteran instructor groups did not differ significantly in terms of students’ self-reported improvement in sketching ability. Also, the two groups did not show a significant difference on two items pertaining to the more technical side of the sketching instruction (enhancing students’ ability to use CAD and improving students’ ability to prepare a set of working drawings for manufacturing). Students in the two groups also provided comparable levels of agreement that the grading of the sketching portion of the course was fair and that they would have preferred to move straight to CAD without spending time on hand sketching.

Significantly higher levels of agreement were reported by veteran instructor group students as compared to control group students for the following aspects/outcomes of the sketching instruction: improved communication with sketches, improved creativity, being a good use of time, increased confidence in sketching ability, serving students well in later engineering courses, usefulness of peer feedback, sketching more frequently in the future, improved visualization skills, and enjoying the sketching instruction. And students in the new instructor group found feedback from their TAs to be significantly more helpful than did students in the veteran instructor group. So students across sections provided favorable opinions about the sketching instruction, but on most aspects, veteran sketching instructor group students provided significantly more favorable opinions than did new instructor group students.

The Fall 2015 data represent two distinct instructional approaches to teaching sketching to students, while the Spring 2016 data represent different instructor experience levels with the same instructional approach. It should be noted that the new instructor’s Spring 2016 implementation included adopting most but not all elements of the redesigned sketching curriculum, so this should be considered a partial rather than a full adoption of the revised curriculum. Reflecting the heightened convergence in the instructional method present during the Spring 2016 semester as compared to the Fall 2015 semester, the student results likewise converged somewhat, in that significantly different (*p* < .05) student perceptions are present in 11/13 (85%) items in the Fall 2015 data and 10/15 (67%) items in the Spring 2016 data. Meaningful differences obviously persist in the new and veteran instructor groups, however, despite the convergence in instructional methods, as student perceptions still differed significantly in the majority of items in the Spring 2016 data.

It should also be noted that the lack of a sketching performance assessment limits our understanding of these differences solely to those related to student perceptions without allowing for an understanding of any differential impacts on empirically evaluated sketching skills. While a sketching performance assessment was not carried out with the full sample, such an assessment was implemented in a related line of research, using a subsample of the Fall 2015 students reported on above. Students from one experimental section and one control section took a sketching quiz at the start and end of the semester. Pre vs. post semester quiz score comparisons revealed that in both conditions, a majority of students improved from the pre to the post, but that experimental group students were significantly more likely to experience an improvement from pre to post than were control group students (Hilton et al. 2017). While these results do not represent the full sample, they provide compelling evidence that while both methods are associated with improved sketching ability for most students, the methods employed in the redesigned curriculum provided this benefit to a larger portion of students when compared to the traditional curriculum, and support the more positive student perceptions of the sketching curriculum reported by experimental group students as compared to control group students.

### End of semester survey

Results for the end of semester survey are shown in Table 2. Across both semesters in which data were collected, survey results indicated that students had generally positive experiences with the CAD instruction, the individual design project, and the team project portions of IEG. Students also reported positive perceptions of the utility of their sketching training throughout the rest of the course. This generally positive feedback is indicated by mean survey responses above 3.5 (which is the midpoint of the strongly disagree to strongly agree response scale used in the survey) for nearly all items in both semesters.

**Table 2:** End of semester survey results (Fall 2015 and Spring 2016)

Survey item	Fall 2015							Spring 2016						
	Control			Experimental			*t* value	New			Veteran			*t* value
	*N*	Mean	SD	*N*	Mean	SD		*N*	Mean	SD	*N*	Mean	SD	
The sketching training was useful throughout the semester.	41	4.39	1.16	95	4.53	0.94	0.72	138	4.36	1.14	170	4.51	1.01	− 1.28
I continued to sketch for this course after the sketching portion of the course was over, even when it was not directly assigned.	40	2.90	1.37	95	3.63	1.33	2.89*	138	3.02	1.39	170	3.07	1.42	− 0.30
The sketching training increased my understanding of what I was doing in CAD.	41	4.15	1.04	95	4.08	1.14	− 0.30	138	3.95	1.36	169	3.82	1.23	0.90
The sketching training increased my ability to determine what was and was not feasible in CAD.	41	3.61	1.28	94	3.56	1.36	− 0.18	138	3.47	1.31	170	3.48	1.20	− 0.04
The sketching training increased my ability to visualize things in CAD.	41	3.98	1.08	95	4.13	1.17	0.71	138	4.16	1.34	170	4.18	1.19	− 0.12
I see more value in the sketching training now that I have learned CAD as compared to before I learned CAD.	41	3.98	1.27	95	4.01	1.12	0.16	138	3.89	1.31	170	3.81	1.26	0.58
The CAD instruction I received in this course was of sufficient length.	41	4.44	1.29	94	4.62	1.00	0.87	138	4.28	1.27	169	4.17	1.10	0.76
The CAD instruction I received in this course covered all of the topics relevant to the course goals.	41	4.80	0.84	94	4.81	0.82	0.02	138	4.60	1.08	170	4.52	0.99	0.66
The CAD instruction I received in this course was useful in helping me learn CAD.	41	5.20	0.51	94	4.68	1.19	− 3.51*	138	4.94	1.01	168	4.60	1.09	2.84*
I feel confident in my ability to use CAD to successfully execute the types of designs covered in this course.	41	5.17	0.67	94	4.94	0.79	− 1.66	138	5.07	0.81	169	4.71	1.02	3.48*
I feel confident in my ability to use CAD moving forward to later courses, internships, and/or co-ops.	41	5.10	0.80	94	4.99	0.91	− 0.66	138	4.86	0.93	170	4.64	1.05	1.96
The CAD instruction I received in this course was sufficiently detailed and thorough for me to feel comfortable with lab assignments.	41	4.44	1.05	92	4.60	1.24	0.71	138	4.38	1.23	169	4.10	1.09	2.06*
The amount of CAD training I was expected to learn on my own was appropriate.	41	4.37	1.20	92	4.34	1.29	− 0.12	138	4.15	1.29	170	4.10	1.14	0.38
I had sufficient access to help from instructors and TAs while I was working on CAD assignments.	41	4.27	1.25	92	4.80	1.00	2.65*	138	4.44	1.25	169	4.42	1.07	0.17
The workload associated with the CAD portion of the course was appropriate.	41	4.37	0.89	92	4.96	0.75	3.71*	138	4.35	1.06	170	4.49	0.93	− 1.24
The CAD instruction sufficiently addressed basic manufacturing processes and vocabulary.	40	5.05	0.75	92	4.65	1.02	− 2.50*	137	4.42	0.98	170	4.63	0.87	− 1.95
The CAD instruction was successful in helping me understand the necessary considerations for manufacturing design.	41	4.83	0.89	92	4.65	0.99	− 0.98	138	4.24	1.06	170	4.49	0.96	− 2.16*
The 3D printing/individual design project was useful.	41	5.15	0.69	91	5.08	0.99	− 0.41	138	4.41	1.06	170	4.76	0.88	− 3.19*
The 3D printing/individual design project was enjoyable.	41	4.68	1.08	91	4.93	1.10	1.22	137	4.07	1.27	170	4.74	1.02	− 5.04*
The format of the 3D printing/individual design project was appropriate.	41	5.07	0.75	91	5.01	0.84	− 0.41	138	4.49	1.01	170	4.72	0.88	− 2.20*
The workload associated with the 3D printing/individual design project was appropriate.	40	4.68	0.92	91	4.96	0.93	1.60	138	4.14	1.14	170	4.76	0.83	− 5.31*
I was satisfied with my level of access to the 3D printers. [experimental sections only]	n/a			91	4.74	1.22	n/a	Due to a survey error, experimental group sections were not asked the experimental section version; their data will be disregarded.						n/a
I would have found it useful to be able to print my individual design project on the 3D printer. [control sections only]	41	4.29	1.52	n/a			n/a	138	4.30	1.37	n/a			n/a
The 3D printing/individual design project increased my understanding of CAD.	41	4.98	0.91	90	4.86	1.13	− 0.60	138	4.65	1.05	170	4.94	0.84	− 2.62*
The 3D printing/individual design project increased my understanding of sketching.	41	4.51	1.19	91	3.95	1.26	− 2.44*	138	3.90	1.30	170	3.71	1.36	1.22
The 3D printing/individual design project reinforced my interest in my major.	41	4.54	1.25	91	4.93	1.03	1.92	138	3.96	1.36	170	4.58	1.19	− 4.26*
The team project was useful.	40	5.18	0.55	91	5.00	0.80	− 1.25	138	4.51	1.11	169	4.62	0.88	− 0.93
The team project was enjoyable.	41	4.49	1.08	91	4.57	1.14	0.40	138	4.08	1.19	170	4.20	1.14	− 0.90
The format of the team project was appropriate.	41	4.85	0.82	91	4.91	0.84	0.37	138	4.40	1.00	169	4.58	0.81	− 1.72
The workload associated with the team project was appropriate.	41	4.46	1.16	91	4.63	1.14	0.75	138	3.94	1.25	169	4.41	0.97	− 3.63*
The team project increased my understanding of CAD.	41	5.22	0.69	91	4.89	1.17	− 2.02	137	4.64	1.06	170	4.60	0.95	0.37
The team project increased my understanding of sketching.	41	4.27	1.30	91	3.88	1.32	− 1.58	138	3.83	1.28	170	3.88	1.23	− 0.39
The team project reinforced my interest in my major.	41	4.71	1.01	91	4.74	1.11	0.14	138	4.12	1.28	170	4.38	1.17	− 1.91

*n/a* not applicable

*Statistical significance of *p* < .05

In Fall 2015, all experimental group mean responses and all but one control group mean responses were above the 3.5 scale midpoint. For the Spring 2016 results, mean responses slightly below the 3.5 response scale midpoint were provided by both student groups on two items: “I continued to sketch for this course after the sketching portion of the course was over, even when it was not directly assigned”, and “The sketching training increased my ability to determine what was and was not feasible in CAD.” It is interesting that the students provided slightly lower mean responses with respect to these two applications of the sketching instruction, regardless of the instructor’s experience level with new sketching methodology.

#### Utility of sketching instruction

For the section on sketching, students were asked about their level of agreement with a variety of statements on the utility of the sketching instruction during the latter, non-sketching focused, portions of the course. The sketching survey data, which are collected immediately after the 6-week sketching portion of the course, revealed that students in the two instructional setting conditions and in the two instructor experience level conditions had largely different perceptions of the sketching instruction itself. But on these items about later utility of the sketching instruction, minimal differences in student perceptions were seen. Overall, these results indicate that, for the most part, these differential experiences during the actual sketching instructions did not translate into differential perceptions of how the sketching instruction served students during the remainder of the course.

#### Utility of CAD instruction

Students provided favorable opinions about the numerous aspects of the CAD instruction and its utility on which they were surveyed. On the majority of items, students reported comparable experiences across conditions. Given the critical nature of an engineer’s ability to create visual representations via both sketching and CAD (Dym et al. 2005), these overall positive student perceptions of the CAD instruction represent a meaningful result, suggesting that the new approach to CAD instruction is an acceptable alternative, at least as far as student perceptions reveal.

Significant differences emerged in student perceptions on roughly 1/3 of items for each semester. In Fall 2015, with respect to two of the more technical aspects of the CAD instruction, its utility in helping students learn CAD and its ability to address basic manufacturing processes and vocabulary, the control group students reported more positive perceptions. On two of the more implementation-oriented aspects of CAD instruction, the appropriateness of the workload and students’ access to help from TAs and instructors, the experimental group students fared better.

In Spring 2016, on three of the more practical and course-related aspects of the CAD instruction, its utility in helping students learn CAD, students’ confidence in their ability to use CAD to execute the designs covered in the course, and the extent to which students felt the CAD instruction was sufficiently detailed and thorough for them to feel comfortable with lab assignments, the new instructor students reported more positive perceptions. On an item related to a broader application of the CAD instruction, its success in helping students understand the necessary considerations for manufacturing design, the veteran instructors’ students’ perceptions were higher than those provided by the new instructor’s students.

#### Perceptions of individual and team projects

Students reported positive perceptions of both the individual and team projects, and these perceptions were similar across conditions, with the exception of the Spring 2016 results on the individual project, which will be discussed later in this section. These projects entailed a high degree of student choice in the items being designed, and were intended to replicate the experience of a real-world design project, to the extent possible in a classroom setting. We feel the favorable ratings of both of these projects align with the literature suggesting that students’ development toward a “practitioner” level of performance is supported by their involvement in authentic tasks (Bhattacharyya and Bodner 2014).

For Fall 2015, the individual project results and team project student responses each included only one significant difference: the control group provided a significantly higher level of agreement with the statements “The 3D printing/individual design project increased my understanding of sketching” and “The team project increased my understanding of CAD”. So while Fall 2015 students provided fairly consistent perceptions of these two projects, it does seem that the control group instructor’s implementation of these projects was slightly more successful in linking it to other portions of the course. The Spring 2016 team project results followed this pattern of similar responses across conditions, with only one significant difference (*p* < .05) being present: The veteran instructor group provided a significantly higher level of agreement with the statement “The workload associated with the team project was appropriate”. Veteran instructors either implemented the team project with a lower workload, or students perceived such a difference.

In a departure from this pattern, the Spring 2016 data included significant differences in six of the seven individual project items, all in the direction of more positive perceptions among the veteran instructor group: the veteran instructor group provided a significantly higher level of agreement with the following statements: “The 3D printing/individual design project was useful”, “The 3D printing/individual design project was enjoyable”, “The format of the 3D printing/individual design project was appropriate”, “The 3D printing/individual design project increased my understanding of CAD”, and “The 3D printing/individual design project reinforced my interest in my major”. Students from the new and veteran instructor groups reported more significantly different experiences with the individual design project than with any other aspect of the course. These findings differ markedly from those seen in the Fall 2015 end of semester survey where a significant difference between control and experimental groups was present on only a single item, suggesting that one or both groups of instructors likely made some changes to their implementation of this project.

## Discussion

The research efforts described in this paper can be discussed at two levels. First, the paper provides insights about the practice of carrying out DBER within the context of investigating a curriculum redesign in a large, introductory level engineering course. Recognizing the importance of using the DBER framework within undergraduate curriculum serves to increase the collaboration between educational researchers and core science (e.g., engineering) scholars. Thus, one significant contribution of this research was to demonstrate the enactment of DBER, which in this case was critical to the successful investigation of the mechanical engineering curriculum redesign. All instructors who taught the class during the period in which this research was conducted were both enthusiastic about learning and adopting the redesigned curriculum, and supported data collection activities during their class time. This is attributable both to the department’s support of the curriculum redesign and our research efforts and to the positive attitudes and openness to learning demonstrated by the instructors of this course. Frequent meetings between the educational researchers and the course instructors were instrumental in both informing the research and instrument design and also in ensuring that the details of the curriculum redesign were well understood by everyone involved in the project. The success of the collaborative aspect of this project also represents a successful outcome of using the DBER framework (National Research Council 2012). By working closely with the course instructors to understand the nature of the content being taught and assessed, the educational researchers gained content knowledge that allowed them to expand the scope of the project to include a separate line of creativity-related research.

Second, the study results provided strong evidence that, while nearly all course components on which students provided feedback were rated positively, the redesigned sketching curriculum components were consistently rated more favorably than were those of the original curriculum. These data suggest that instructors can adopt the redesigned sketching curriculum with a reasonable expectation that students might find the new approach equally or more effective than the original approach.

Lack of time to plan and carry out additional research related activities was the only major limiting factor in our ability to fully maximize the outcomes of the project. Instructors used a variety of performance assessments, quizzes, homework assignments, etc., so there was no existing universal task appropriate for a sketching performance assessment across all students. This was recognized as a limitation of our research design, but instructors were not able to develop, administer, and score an additional performance assessment. Additionally, in order to be useful for research purposes, such an assessment would have been quite lengthy, which might result in excess work for students.

Despite these limitations, we feel the project was a success and provides a meaningful contribution to both the DBER and engineering education bodies of literature. We were able to provide evidence to course instructors that the redesigned curriculum is regarded positively by students, and also demonstrated factors that contribute to success in a DBER project.

## Conclusions

The main conclusion of this study is that students reacted positively to the redesigned sketching curriculum, providing instructors some measure of confidence in adopting the new curriculum. With regard to implications for future research, we would urge researchers undertaking similar work to establish and maintain a close working relationship with the instructors implementing the curriculum through the duration of the project. This work also emphasized that, for DBER projects especially, having team members with a high degree of disciplinary content expertise is critical for success. More specifically related to investigating the learning associated with the curriculum, we would advise attempting to include a performance assessment during the early planning stages of the research. Our work relies primarily on student perception data, and while this is highly useful for addressing our research questions, we missed an opportunity to investigate the impact of the redesigned curriculum on students’ sketching ability.

The enactment of DBER via a partnership between education researchers and mechanical engineering faculty was a success due in part to the enthusiastic participation of the faculty members in both the research efforts and in implementing the redesigned course, as well as the support of the faculty members’ department. The combination of expertise in both engineering design and sketching content as well as principles and research methods from educational psychology present among members of the research team lent the research a level of sophistication and nuance that we feel would not have been achieved otherwise. Adopting the DBER framework allowed us to glean a deep and detailed understanding of how entry level engineering students react to various aspects of the sketching instruction they received, and furthermore, how these reactions vary on the basis of both instructional method and instructor experience with the curriculum. We did not simply gain a deeper understanding of how students react to a general instructional strategy, but rather we learned how they react to being taught a highly discipline-specific topic in a highly discipline-specific manner.

## Abbreviations

AE: Aerospace engineering
CAD: Computer-aided design
DBER: Discipline-based education research
EER: Engineering education research
GD&T: Geometric dimensioning and tolerancing
IEG: Introduction to Engineering Graphics
ME: Mechanical engineering
NRC: National Research Council
NSF: National Science Foundation
RREE: Rigorous research in engineering education
STEM: Science, technology, engineering, and math
